# Identification, Characterization, and Ultrastructure Analysis of the Phenol-Degrading *Rhodococcus erythropolis* 7Ba and Its Viable but Nonculturable Forms

**DOI:** 10.3390/microorganisms12122662

**Published:** 2024-12-22

**Authors:** Valentina N. Polivtseva, Anton N. Zvonarev, Olesya I. Sazonova, Yanina A. Delegan, Yulia N. Kocharovskaya, Alexander G. Bogun, Nataliya E. Suzina

**Affiliations:** 1 Institute of Biochemistry and Physiology of Microorganisms, Federal Research Center “Pushchino Scientific Center for Biological Research of Russian Academy of Sciences” (FRC PSCBR RAS), 142290 Pushchino, Russia; zvonarevibpm@gmail.com (A.N.Z.); sazonova_oi@rambler.ru (O.I.S.); mewgia@yandex.ru (Y.A.D.); kocharovskayaj@mail.ru (Y.N.K.); bogun62@mail.ru (A.G.B.); suzina_nataliya@rambler.ru (N.E.S.); 2 Academy of Biology and Biotechnology Behalf D.I. Ivanovskyi, Southern Federal University, 344006 Rostov-on-Don, Russia

**Keywords:** biodegradation, VBNC, *Rhodococcus*, phenol, ultrastructural cell organization, Rpf, long-term storage

## Abstract

Phenol and its chlorinated derivatives are introduced into the environment with wastewater effluents from various industries, becoming toxic pollutants. Phenol-degrading bacteria are important objects of research; among them, representatives of the genus *Rhodoccocus* are often highlighted as promising. Strain 7Ba was isolated by enrichment culture. A new isolate was characterized using culturing, biochemistry, high-throughput sequencing, microscopy (including electron microscopy), and functional genome analysis. *Rhodococcus erythropolis* strain 7Ba is able to grow on phenol and chlorophenols without losing its properties during long-term storage. It was shown that strain 7Ba is able to form viable but nonculturable (VBNC) forms during long-term storage under nutrient limitation, preserving both cell viability and the ability to degrade phenols. The ultrastructural organization of the vegetative forms of cells and VBNC forms was characterized. The following distinctive features were found: modifications (thickening) of cell membranes, cell size reduction, nucleoid condensation. Functional analysis of the genome showed the presence of genes for the degradation of alkanes, and two branches of the β-ketoadipate pathway for the degradation of aromatic compounds. Also, the genome of strain 7Ba contains several copies of Rpf (resuscitation promoting factor) genes, a resuscitation factor of resting bacterial forms. The new isolate strain 7Ba is a promising biotechnological agent that can not only utilize toxic aromatic compounds but also remain viable during long-term storage. For this reason, its further application as an agent for bioremediation can be successful under changing conditions of climate and given the deficiency of nutrient compounds in nature. Minor biostimulation will allow the strain to recover its metabolic activity and effectively degrade pollution.

## 1. Introduction

Bioremediation is an efficient, cost-effective, and safe way to treat the anthropogenically polluted environment [1,2]. Physical and chemical methods of cleaning have been used for a very long time; they are effective, but they cause additional harm to the environment. This is due to the use of various chemical compounds and the physical impact on nature, which changes it, destroying the established interrelationships of living organisms [3,4]. In this regard, the search for biological agents capable of degrading toxic compounds is relevant, and the importance of new isolates is not lost. One of the common anthropogenic pollutants are phenols (phenol, its derivatives). Phenols enter surface waters with wastewater from oil refining, coke-chemical, pulp, and paper industries and occupy one of the leading positions in terms of emissions of environmental pollutants [3]. They are a raw material for the pharmaceutical industry, production of phenol-formaldehyde resins, and others [3,5,6]. Microorganisms are capable of degrading these compounds to safe end products: carbon dioxide and water. Among the microorganisms that degrade phenolic compounds are bacteria, algae, and fungi [7,8,9,10,11].

Due to the ability of microorganisms to live in various environments, including constantly changing factors such as temperature, pH, salinity, etc., as well as their ability to degrade a wide range of pollutants, they are a universal object of remediation. Despite this, there are a number of issues that require further research; for example, the question of resistance of bacteria destructors to the effects of high concentrations of pollutants, and the study of the possibility of stimulating the life activity of microorganisms-destructors directly inhabiting the territory for bioremediation; in other cases, it is necessary to strictly control the introduction of new bioagents into the environment in order not to break the ecological relationships among the indigenous population [12,13,14].

However, biodegradation efficiency may be limited in part because bacteria tend to enter an inactive state. A common inactive state of nonsporulating bacteria is the viable but nonculturable (VBNC) state, in which cells exhibit significantly less metabolic activity than their actively growing counterparts [15,16]. The VBNC state is an adaptive strategy for non-spore-forming bacteria in which cells remain viable with low metabolic activity, low respiratory activity, and intact cell membranes, but are unable to grow and divide on conventional media [17,18].

To date, more than a hundred species of microorganisms have been shown to enter the VBNC state under various stress conditions, including a high concentration of pollutants, extreme temperature, osmotic pressure, oligotrophy, hypoxia, and other physicochemical stresses [19,20,21]. In particular, the VBNC state has been identified in strains capable of degrading biphenyl, PCBs, phenol, 4-chlorophenol, and polycyclic aromatic hydrocarbons (PAHs) [15,18,22,23,24].

Although direct elimination of stressors such as elevated temperature [25] and adequate nutrient availability [26] can return VBNC bacteria to a state suitable for culturing, an important discovery was the identification in the 1990s of resuscitation-promoting factor (Rpf), a secretory protein in the supernatant of actively growing *Micrococcus luteus* [27,28]. Rpf acts on the bacterial cell wall, degrades the cell wall peptidoglycan of VBNC cells, and triggers reanimation [29,30,31].

Orthologs of the Rpf gene encoding Rpf-like proteins have been identified among various Gram-positive (GC%)-rich bacteria, including representatives of the genera *Mycobacterium*, *Corynebacterium*, *Nocardia*, and *Rhodococcus* [32,33,34]. Studies have shown that Rpf from M. luteus and other Rpf homologs can reanimate VBNC cells of both Gram-positive and Gram-negative bacteria, including *Mycobacterium*, *Rhodococcus*, *Corynebacterium*, *Nocardia*, *Achromobacter*, *Arthrobacter*, *Bacillus*, *Alcaligenes*, and *Pseudomonas* [35,36,37].

When favorable conditions occur, bacteria become active and are again capable of the degradation of pollutants. In this case, different authors observed an increase in metabolic activity, including an increase in the spectrum of substrates for degradation. For the strain *R. opacus* 1CP, it was found that after resuscitation from dormancy the strain became able to utilize a number of di- and trichlorophenols that were not previously growth substrates of 1CP [38]. Ye’s group found that addition of the reactivation factor Rpf enhanced biodegradation of biphenyls by *Rhodococcus biphenylivorans* TG9T [39]. Su’s group also showed that addition of Rpf enhanced PVC degradation in soil [22,40]. Xinru Zhou’s group showed that addition of the reactivation factor and/or reanimated strain results in enhanced degradation of polyvinyl chloride in soil [41]. The addition of recombinant Rpf2 from *Rhodococcus erythropolis* increases the efficiency of oil degradation by indigenous microorganisms in the soil, and the number of culturable bacterial groups is increased when isolated from contaminated soil [42].

The aim of our work was to isolate a new strain capable of degrading phenol, to study its ability to survive unfavorable conditions with the formation of the VBNC stage while retaining degradative activity after leaving the VBNC state.

## 2. Materials and Methods

### 2.1. Organisms, Methods of Cultivation

The soil near the territory of the oil refinery (Saratov, “Saratov Oil Refinery”, Russia) was the material for isolation of pollutant-degrading bacteria. The soil in the sampling area was contaminated with oil sludge and heavy metals in trace amounts. Sampling was carried out from a depth of 5–10 cm from several points. Samples were taken in the sterile bags with a Ziploc. Total soil was used for isolation of microorganisms after mixing the collected samples. Soil samples (5 g) were introduced into Erlenmeyer flasks containing 100 mL of mineral medium (composition, g/L: Na_2_HPO_4_—0.7; KH_2_PO_4_—0.5; NH_4_NO_3_—0.75; MgSO_4_ × 7H_2_O—0.2; MnSO_4_—0.001; FeSO_4_—0.02, NaHCO_3_—0.25) containing 5% (vol/vol) meat-peptone medium (LB) as growth substrate. The flasks were cultured on a shaker (180 rpm) at 28 °C for 7 days. Cells were cultured on solid rich medium after dilution to 10^–6^–10^–8^. Individual colonies differing in morphotype were seeded on rich medium for further work. Strains from the obtained collection (18 isolates) were tested in the first step for their ability to degrade phenol at a concentration of 0.2 g/L. Isolate 7Ba was selected in this way.

### 2.2. Biochemical Tests

Biochemical properties of the isolate were analyzed using IP 32E, 50CH, and API 20E tests (Biomerieux, Marcy-l’Étoile, France).

### 2.3. Determination of Bacterial Degradation Ability

The 7Ba isolate obtained from the enrichment culture was tested for its ability to utilize various phenolic compounds, which were added to the mineral medium as the sole source of carbon and energy. Growth substrates were used in the following concentrations: phenol 0.2–1.0 g/L; benzoate and hexadecane in concentrations up to 0.1 g/L, chlorophenols (2-, 2,4-, 2,4,6-trichlorophenol)—0.1 g/L. Culture growth was monitored by measuring the optical density (OD) at 560 nm, phenol loss was determined by a spectrum in the range of 220–350 nm (Shimadzu, Japan), pH of the medium was maintained in the range of 7.0–7.2 by adding NaOH.

### 2.4. Microscopic Investigations

#### 2.4.1. Light Microscopy

Microscopic observations were performed using Nikon Eclipse Ci microscope (Nikon, Tokyo, Japan) with ProgRes SpeedXT camera (Jenoptic, Jena, Germany).

Live and dead cells were detected using the LIVE/DEAD™ BacLight™ Bacterial Viability Kit (Molecular Probes, Eugene, OR, USA). First, 1 µL of the relevant dye from the kit was added to 0.5 mL of microbial suspension (cell concentration was 10^6^–10^7^ CFU/mL). Samples were examined by fluorescence microscopy on an AXIO Imager A1 instrument (Zeiss, Jena, Germany) with a 56HE filter set (Zeiss, Germany) at 470 nm (excitation) and 512 nm + 630 nm (emission). Axiocam 506 camera (Zeiss, Germany) was used for obtaining images.

#### 2.4.2. Transmission Electron Microscopy

The preparation of intact bacterial cells was negatively stained with 0.2% uranyl acetate solution. To prepare ultrathin sections, cells of the tested variants, including VBNC forms, were centrifuged (10,000× *g*, 15 min) and incubated in 2% glutaraldehyde solution in 0.05 cacodylate buffer (pH 7.2) for 1 h at 4 °C. After three cycles of washing the samples in 0.05 cacodylate buffer (pH 7.2), they were fixed with a solution of 2% OsO4 in the same buffer and left for 4 h at 18–20 °C. Dehydrated samples were then embedded in Epon 812 epoxy resin. According to Reynolds [43], ultrathin sections were sequentially contrasted for 30 min in a 3% uranyl acetate solution in 70% alcohol and 4 min with lead citrate. A transmission electron microscope JEM-1400 (JEOL, Tokyo, Japan) at an accelerating voltage of 80 kV was used to study ultrathin sections and negatively stained preparations.

### 2.5. Preparation of Cell-Free Extract

To obtain biomass, the strains were cultured on mineral medium, and the cultures were inoculated into 750 mL flasks containing 200 mL each of mineral medium supplemented with phenol (0.2 g/L) as the sole source of carbon and energy. The bacteria were grown on a shaker at 220 rpm at 28 °C to an optical density of 0.7–0.8 optic units, then phenol (0.2 g/L) was reintroduced as it was consumed by the culture.

The cell-free extract was prepared as described in [44]. Briefly, cells were disrupted by extrusion disintegration on a Hughes-type press (IBPM RAS, Moscow, Russia), then centrifuged at 10,000× *g* (4 °C, 30 min) in the presence of trace amounts of DNAase. The supernatant was used as cell-free extract for determination of enzyme activities. Then, 5–50 µL of extract was added to 1.0 mL of reaction mixture. The activities were determined at 25 °C, the reaction was started by adding the cell-free extract, and the OD was determined on a UV-1800 spectrophotometer (Shimadzu, Tokyo, Japan).

### 2.6. Enzyme Activity

The activities of the key enzymes of aromatic ring degradation—catechol-1,2-oxygenase, catechol-2,3-oxygenase, protocatechuate-3,4-dioxygenase, muconatecycloisomerase, and gentisate-1,2-dioxygenase—were determined according to Egozarian et al. 2024 [45].

Micromoles of substrate consumed or product formed per minute per 1 mg of total bacterial protein were used to express specific enzyme activities. By using the Bradford method, the concentration of proteins was determined through spectrophotometric measurements [46].

### 2.7. Obtaining VBNC Cells

Cells of strain 7Ba were induced into the VBNC state by nutrient and temperature limitation. The first method was to use rich medium, by growing the cells in a flask with LB medium for 3 days at 28 °C on a shaker to maximize nutrient limitation. The flask was then placed in a box and stored in the dark at 24 °C for 1.5 years. After 1.5 years, a sample was taken from the flask for further studies and the remaining cell biomass in VBNC condition continued to be stored at 4 °C. After another two years (total storage time was 3.5 years), the cell biomass was again sampled for research. The second method consisted of storage on agarized rich LB medium at 4 °C for 4–6 months. In this case, the culture dried out and needed to be hydrated with sterile tap water/nutrient medium before working with it. VBNC sampling was performed under aseptic conditions.

### 2.8. Determination of Cell Viability

The colony-forming unit (CFU) method was used to determine the number of cells capable of resuscitation from the VBNC state. Briefly, VBNC cells in different dilutions were plated on petri dishes with LB nutrient medium and cultured for 3 days at 28 °C. Colony counting was performed on a colony counter SC6 (Stuart, Inverness, UK) and cell numbers were expressed in CFU/mL.

### 2.9. DNA Sequencing, Assembly, and Annotation of Complete Genomes

The genome of strain 7Ba was sequenced on DNBSEQ-G50 (MGISEQ-200) equipment (MGI, Wuhan, China). It generated 1,349,672 paired-end reads of 150 bp in length. Data filtering was performed with Trimmomatic v. 0.39 using the following parameters: LEADING:15 TRAILING:15 SLIDINGWINDOW:4:15 MINLEN:100. The filtering results are presented in the Table 1.

Assembly was performed using SPAdes v. 3.15.4. Contigs shorter than 500 bp were removed. To assess the quality of the genome, CheckM v. 1.2.2 [47] was used. Genome annotation was performed using Prokka v. 1.14.5 and RAST (https://rast.nmpdr.org/, accessed on 15 May 2023). The genome of strain 7Ba was annotated in GenBank: BioSample ID SAMN40030130, BioProject number PRJNA1079062, WGS project—JBAKBV01. The whole-genome tree was built using the TYGS web service [https://tygs.dsmz.de/ (accessed 25 November 2024)] from Genome BLAST Distance Phylogeny (GBDP) distances using “greedy-with-trimming” algorithm. The algorithm “greedy-with-trimming” involves removing the overlapping parts of high-scoring segment pairs in either genome [48]. The tree was built using only the genomes of type strains of relative species. GBDP defines distances between pairs of fully or partially sequenced genomes. The ANI value was calculated using the EzBioCloud ANI Calculator [49]. DNA-DNA hybridization (DDH) was calculated using the Genome-to-Genome Distance Calculator (GGDC) [48]. Functional annotation of the genome was performed using KEGG [50]. MGE search was performed using Mobile-OG db [51]. Alien_Hunter [52] was used to detect horizontally transferred regions in the genome.

### 2.10. Genomic Fingerprinting

Two genomic fingerprints were performed to establish genotypic differences between the studied samples. The rep-PCR (repetitive sequence-based PCR) used either primer (GTG)5 (5′-GTGGGGTGGTGGTGGTGGTGGTGGTGGTGGTG-3′) or Box A1R (5′-CTACGGCAAGGCGCGACGCTGACGACG-3′) [53], and the amplification program according to Mohapatra et al. [54]. The reaction was conducted in a sample of 25 μL. The composition of the PCR mixture included the following: 100 ng of DNA matrix, 2 μM primer, 1 × PCR buffer, 200 μM deoxyribonucleoside triphosphate, 3 mM MgCl_2_, 5% dimethyl sulfoxide (DMSO), 0.1 mg/mL bovine serum albumin, and 2.5 units of DreamTaq polymerase. The polymerase chain reaction (PCR) was conducted using a GeneAmp PCR System 9700 amplifier (Applied Biosystems, USA). Using a 1.2% horizontal agarose gel and 0.5x Tris-borate buffer, electrophoresis was carried out [55]. By staining the gel with ethidium bromide solution, we were able to visualize DNA. For the sample plating, we used a buffer of 0.025% xylolcyanol, 0.025% bromophenol blue, 2.5% ficol (type 400). The standard GeneRuler 1 kb Plus DNA Ladder was employed as the marker DNA. It documented the gel on a BioTestColor system (Moscow, Russia).

### 2.11. Statistical Analysis

All studies were performed with a minimum of three repetitions. All statistical procedures were conducted with SPSS software version 23.0 (IBM Corporation, New York, NY, USA) and data statistical analysis package of Microsoft Excel 2016 (Microsoft, Redmond, WA, USA). Differences were tested by independent-sample *t*-test or one-way analysis of variance. A significance level of *p* ≤ 0.05 was set.

## 3. Results

### 3.1. Isolation, Morphology, and Biochemical Properties

By the method of enrichment cultures, strain 7Ba was isolated from contaminated soil (Saratov, “Saratov Oil Refinery”, Russia). The cells of isolate 7Ba have the appearance of long branching immobile bacilli, characteristic in morphology of representatives of the genus *Rhodococcus*. Colonies have a beige color, and are smooth with even edges.

Analysis of biochemical properties by tests showed that strain 7Ba is urease-positive, able to utilize citrate, has lipase, β-glucuronidase, and β-galactosidase activities, does not reduce nitrate, and is able to oxidize D-fructose and L-arabinose. It does not oxidize a large number of organic compounds: D-glucose, D-mannitol, inositol, D-sorbitol, L-rhamnose, D-saccharose, D-melibiose, amygdalin, and does not liquefy gelatin.

### 3.2. Phenol Degradation Ability Before and After Recovery from the VBNC State

**Figure 1:**
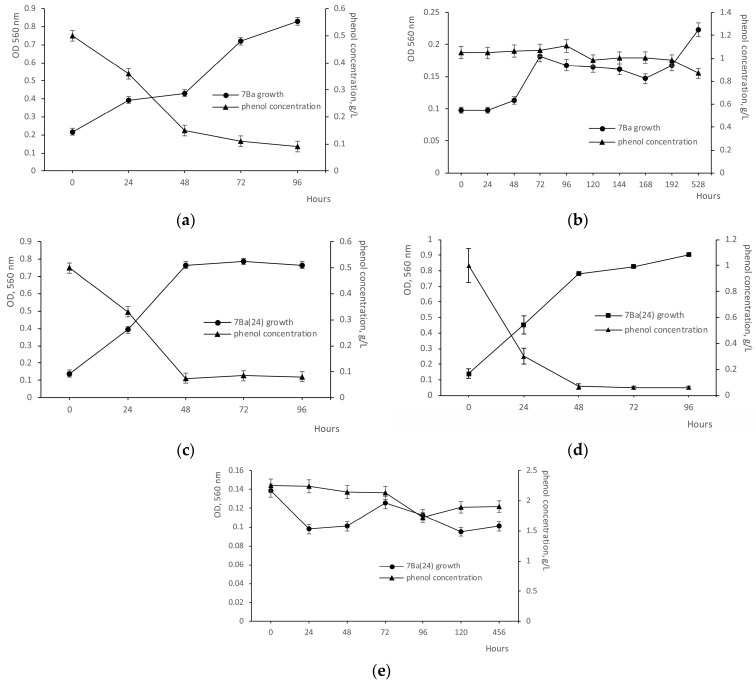
Growth of strain 7Ba on phenol: (**a**) vegetative cells 7Ba from the reseeded culture, phenol 0.5 g/L, (**b**) vegetative cells 7Ba from the reseeded culture, phenol 1 g/L, (**c**) 7Ba(24) cells (vegetative cells of strain 7Ba after dormancy at 24 °C), phenol 0.5 g/L, (**d**) 7Ba(24) cells (vegetative cells of strain 7Ba after dormancy at 24 °C), phenol 1 g/L, (**e**) 7Ba(24) cells (vegetative cells of strain 7Ba after dormancy at 24 °C), phenol up to 2 g/L.

**Figure 2:**
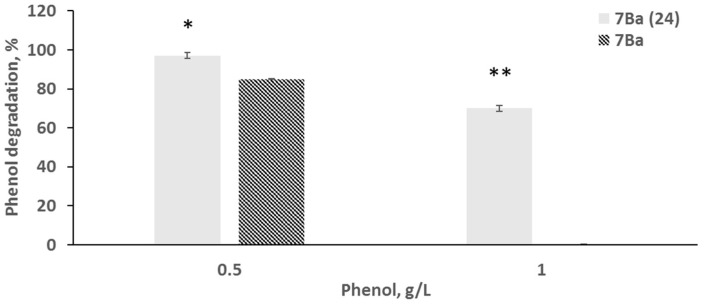
Degradation level of phenol by 7Ba and 7Ba (24). Values are mean ± SD of triplicate sets, * *p*-value ≤ 0.05 and ** *p*-value = less than 0.001 represent the significant difference between variants.

Analysis of growth on medium with different concentrations of phenol showed that strain 7Ba was able to grow and degrade phenol at concentrations up to 0.5 g/L. The results of substrate consumption control showed that 70% of phenol was utilized on the second day, and 82% on the 4th day, with growth slowing down on the fourth day and requiring phenol addition for activation (Figure 1a and Figure 2). Growth at 1 g/L resulted in a small increase in biomass, practically without a decrease in substrate concentration (Figure 1b).

The cleavage of the benzene ring of phenols can be performed by two pathways—meta- and ortho-pathways—which are realized by the enzymes catechol-2,3-oxygenase and catechol-1,2-oxygenase, respectively. Representatives of the genus *Rhodococcus* show the enzyme activity of both pathways for the degradation of phenolic compounds. Therefore, in our study, we analyzed the activity of both enzymes. Analysis of the activity of key enzymes involved in benzene ring cleavage showed that strain 7Ba possesses the activity of enzymes performing degradation of benzene compounds via the β-ketoadipate pathway both through catechol and protocatecholic acid. The activity of the second branch of enzymes, protocatechuate-3,4-dioxygenase, was 14-fold higher than catechol-1,2-oxygenase, reaching 2.202 U/mg protein and 0.150 U/mg protein, respectively. Minor activity of muconatecycloisomerase was also detected, amounting to 0.065 U/mg protein.

We performed experiments on introducing cells of strain 7Ba into the dormant (VBNC) state by two methods: long-term storage (up to 1.5 years) at 24 °C (designated as 7Ba(24)) and storage for 4–6 months at 4 °C (designated as 7Ba(4)). Remarkably, in both cases, cells of strain 7Ba rapidly returned to a metabolically active state: active cell growth was observed by 2 days of VBNC cultivation on rich solid medium. When the grown cells were transferred to mineral medium with phenol 0.2 g/L, an increase in the optical density of cultures and a decrease in phenol concentration were observed. Moreover, in the case of variant 7Ba(24) after 1 day, the phenol concentration decreased almost twofold, while for variant 7Ba(4), a significant decrease in phenol concentration was detected at 2 days.

Further transfer into flasks with mineral medium and phenol at concentrations of 0.5 (Figure 1b) and 1 g/L (Figure 1c) showed that strain 7Ba(24) not only did not lose its ability to grow at high phenol concentrations, but also showed active growth at 1 g/L phenol: after 48 h cultivation, 97% of the substrate was consumed, compared to 0.5 g/L phenol, when after two days, 85% of the substrate was lost (Figure 2). This different rate is probably due to the fact that the enzymes involved in phenol utilization are inducible, and the inoculum for growth at 1 g/L was the material from the culture after 2 days of growth at 0.5 g/L.

No cell growth of strain 7Ba(24) was observed at phenol concentrations up to 2 g/L (Figure 1e). The ability to grow on 1 g/L phenol, obtained by strain 7Ba after the dormancy period, was lost gradually, and after 3–4 reseeding, the culture was capable of utilizing only 0.5 g/L again. Cells of strain 7Ba(24) returned to the physiology that characterized it before VBNC (Figure 1a,b).

The variant strain 7Ba(4), in turn, was unable to grow at a phenol concentration of 1 g/L, as the vegetative cells were routinely reseeded. Further studies focused on 7Ba(24) cells as the most interesting ones that showed adaptation changes related to the VBNC state.

### 3.3. Growth on Toxic Substrates Before and After Dormancy

Examination of the ability to grow on various substrates showed that strain 7Ba utilized phenol (up to 0.5 g/L), benzoate (up to 0.5 g/L), and hexadecane (up to 0.1 g/L).

After dormancy, strain 7Ba(24) not only retained the ability to grow on the previously used toxic single substrates, but also exhibited the ability to grow on phenol chlorine derivatives such as 4-chlorophenol, 2,4-dichlorophenol, and 2,4,6-trichlorophenol at concentrations up to 0.1 g/L, see Table 2.

### 3.4. Microscopy of Vegetative and VBNC Cells

**Figure 3:**
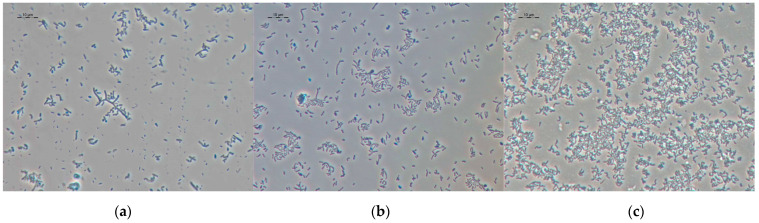
Cell morphology of strain 7Ba: (**a**) vegetative cells 7Ba from the reseeded culture grown on LB medium for 48 h, (**b**) VBNC cells of the 7Ba(4), (**c**) VBNC cells of the 7Ba(24). Phase-contrast microscopy. Scale bar—10 μm.

Vegetative cells of strain 7Ba are polymorphic; both short ovoids up to 0.5 μm and long “branching” sticks up to 20 μm long can be found in the population (Figure 3a). The morphology of cells in the VBNC state is different: cells have more condensed cytoplasm, and long branching cells are practically absent (Figure 3b,c).

**Figure 4:**
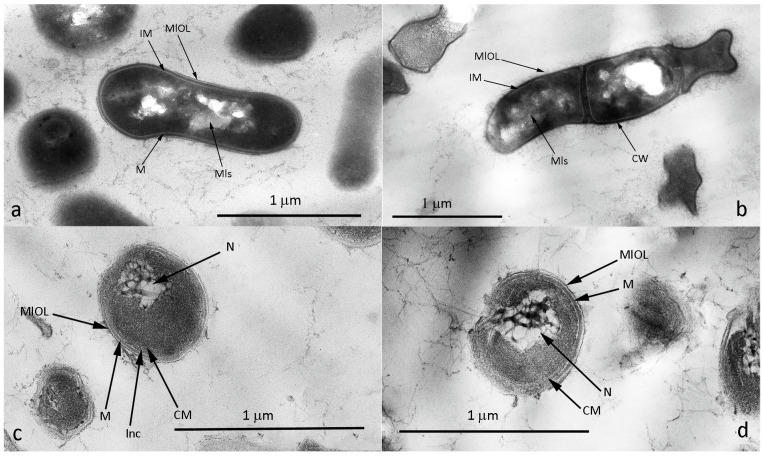
Ultrastructural organization of cells of strain 7Ba: (**a**) vegetative cells 7Ba from the reseeded culture, (**b**) VBNC cells of 7Ba(4), (**c**) VBNC 7Ba(24), (**d**) VBNC 7Ba(24) after 3.5 years of storage. Transmission electron microscopy. Designations: CM—cytoplasm membrane, CW—cell wall, Inc—inclusions, M—murein, MlOL—“membrane-like limiting outer layer”, Mls—myeline-like structures, N—nucleoid, IM—inner space between cytoplasm membrane and peptidoglycan layer.

Analysis of ultrathin sections showed that the vegetative cells of strain 7Ba have a Gram-positive cell wall structure, with the space between the cytoplasmic membrane and the peptidoglycan layer clearly visible. On the outer surface of the peptidoglycan layer, a layer that we have named the “membrane-like limiting outer layer” is clearly distinguishable (Figure 4a). This layer is a phospholipid formation, which can be indirectly evidenced by its detection on cell sections contrasted with osmium tetroxide, which stains protein compounds.

Representatives of the genus *Rhodococcus* in relation to Gram staining belong to the group of Gram-stain-positive to Gram-stain-variable cells [56]. It is probably the presence of this membrane-like layer that determines the variability of staining. The cytoplasm of cells is homogeneous, with inclusions of triacylglyceride and polyphosphate nature, and myeline-like structures characteristic of representatives of this genus are often revealed in the cells [57]. VBNC cells of strain 7Ba(4) differ from vegetative cells by a weakly expressed space between the cytoplasm and the peptidoglycan layer, as well as by loosening and enlargement of the outer layer around the peptidoglycan (Figure 4b). The loosening is probably exactly the “membrane-like restrictive outer layer”, which is poorly detectable in VBNC cells.

VBNC cells of 7Ba(24) after 1.5 (Figure 4c) and 3.5 (Figure 4d) years of storage differ from VBNC cells of 7Ba(4); they show a strongly visible inner space between the cytoplasm membrane and peptidoglycan layer, and the outer MlOL in some places comes off in pieces. The nucleotide condenses and its filaments become highly expressed, taking on the look of a net. Interestingly, no intracellular inclusions are observed in the cytoplasm. But in the formed inner space between the cytoplasm membrane and peptidoglycan layer (murein), inclusions of unclear nature are detected. These inclusions probably have a phospholipid composition and are produced from the cytoplasmic membrane. Thus, the cell forms an additional layer to protect the cytoplasmic contents.

### 3.5. Determination of Cell Viability

**Figure 5:**
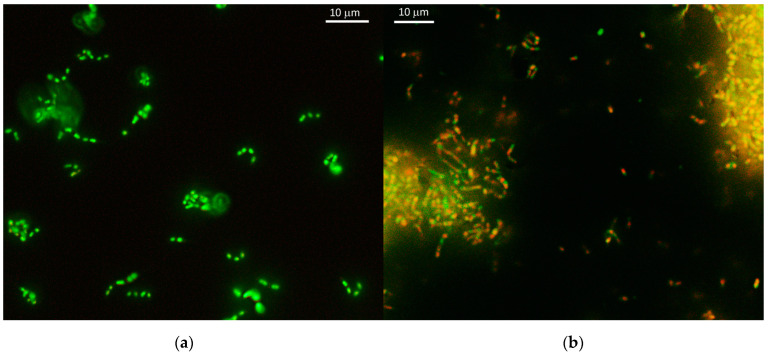
Cells of strain 7Ba stained with Live/Dead dye. (**a**) vegetative cells 7Ba from the reseeded culture, (**b**) VBNC cells of the 7Ba(24) after 3.5 years of storage. Cells that are green in color are live cells, dead cells are red in color. Fluorescence microscopy.

Fluorescence microscopy data show that the cells remain alive after 3.5 years of storage. In the population, we detect both green staining (live cells, Figure 5a) and red staining (dead cells, Figure 5b). In addition, a mixed coloration effect is also observed, where cells become yellow colored. This effect is probably related to the permeabilization of membranes and consequently changes in its permeability to the dye, but does not affect viability.

Rhodococci are capable of forming cellular forms in the shape of long sticks that can divide into smaller ones. In VBNC strain 7Ba(24), both small cocci and bacilli that have not completed division are detected. Live/Dead staining reveals nucleoids stained both green and red in one long cell that has not completed division.

Cultivation of VBNC 7Ba(24) cells after 3.5 years of storage showed that the dormant biomass contained 1.9 ± 0.2 × 10^4^ CFU/mL of live cells, which correlated with fluorescence microscopy data.

### 3.6. Whole Genome, Analysis and Gene Search of Major Degradation Enzymes and Rpf Protein

The genome of strain 7Ba was assembled to contigs, and the assembly metrics are presented in Table 3. Sequencing completeness was 99.94%, and contamination was 0.79%.

**Figure 6:**
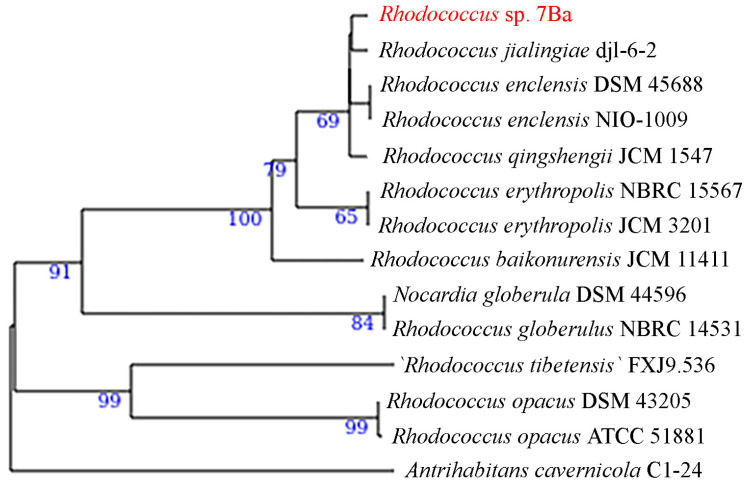
Phylogenetic tree showing the position of strain 7Ba within the genus *Rhodococcus*. The genomes of only type strains were used in the construction of the tree. The strain under study is labeled in red.

On the phylogenetic tree of complete genomes (Figure 6), strain 7Ba is in a clade consisting of members of species synonymous with *R. qingshengii*.

The ANI value of 96% was considered to be the threshold value for species assignment, according to [58]. The DDH threshold was considered to be 70%. This DDH value is the “gold standard” for species delimitation [48]. It should be clarified, however, that *R. qingshengii* is not recognized by modern systematics as a separate species, and therefore it is not possible to distinguish between *R. erythropolis* and *R. erythropolis* (*qingshengii*) using parameters for comparing complete genomes. Strain 7Ba was assigned to *R. erythropolis* (*qingshengii*) based on the data on the number of *alkB* alkan monooxygenase genes (the strain has five genes, whereas *R. erythropolis* has four genes).

The genome contains 6275 CDSs, of which 6225 are protein-coding. There are 61 RNA genes, of which 54 are tRNAs, one complete cluster (5S, 16S, 23S) and one additional copy of 23S are rRNAs, and three are ncRNAs. When the genome was functionally annotated, 42.2% of its protein-coding genes were annotated.

The category “Xenobiotics biodegradation and metabolism” is represented by 68 genes, including genes for the catabolism of benzoate (28 genes) and its various derivatives—aminobenzoates (8) and fluorobenzoates (10)—as well as the transformation of steroidal compounds (13 genes).

In silico genome analysis showed that the genome of strain 7Ba contains genes responsible for the degradation of a number of aromatic and aliphatic compounds. We found clusters of genes responsible for the degradation of alkanes. Previously, in the genomes of already described strains of the genus *Rhodococcus*, the *alkB* gene was usually located in a cluster with genes encoding rubredoxin (*alkG1*, *alkG2* or *rubA1*, *rubA2*) or rubredoxin reductase (*alkT* or *rubB*) [59,60]. In the genome of R. qingshengii, 7Ba has two clusters containing two alkan -1 -monooxygenase genes (EC 1.14.15.3) and four rubredoxin genes. Three separately located *alkB* genes, eight copies of the cytochrome P450 gene, and two copies of alkansulfonate monooxygenase (EC 1.14.14.5) were also detected.

Representatives of the genus *Rhodococcus* are efficient degraders of aromatic compounds; genome analysis of a number of strains of this genus shows the presence of genes of both central and peripheral pathways for the degradation of aromatic compounds [60,61,62,63]. The central pathways of aromatic compound degradation in strain 7Ba are represented by genes of enzymes of two pathways: β-ketoadipate and homogentisate. The β-ketoadipate pathway is represented by two branches, the catechol branch of the β-ketoadipate pathway and the protocatechuate branch of the beta-ketoadipate pathway. The full catechol ortho-cleavage pathway is represented, and three of the five catechol meta-cleavage genes are also detected. The phenylacetate pathway is also incomplete, with six out of seven genes represented according to BLAST KEGG analysis. Of the peripheral pathways for the degradation of aromatic compounds, a cluster of genes encoding proteins for the degradation of benzoate, biphenyl *bphC*, toluene, and xylene are present in the 7Ba genome.

One of the responses to stress during growth on pollutants may be the synthesis of biosurfactants that facilitate access to hydrophobic substrates. The genome of strain 7Ba contains genes—*otsA* (trehalose-6-phosphate synthase), *otsB* (trehalose-6-phosphate phosphatase), and *treZ* (malto-oligosyltrehalose trehalohydrolase)—encoding proteins of surfactant biosynthesis.

Genome analysis of strain 7Ba showed that it contains Rpf protein synthesis genes *rpfA* (1 variant), *rpfA* (2 repeats), and *rpfE* (3 repeats) after analysis with KEGG. RAST annotation detected the genes *rpfA* (1 variant) and *rpfA* (2 repeats). Rpf-encoding genes are widely distributed in *Rhodococcus*. Previously, Yu’s group, analyzing the pangenome of the genus *Rhodococcus*, showed that the gene cluster annotated as *rpfB* is present in 82.7% of the genomes of members of this genus. For *Rhodococcus biphenylivorans* strain TG9, they showed *rpfA*, *rpfB*, *rpfD*, and *rpfE* genes in the whole genome [64].

### 3.7. Genomic Fingerprinting

The rep-PCR method was used to detect possible rearrangements in the genome of a strain of *Rhodococcus* sp. 7Ba. Vegetative cells of strain 7Ba routinely reseeded into laboratory culture, vegetative cells of strain 7Ba after dormancy in two variants, 7Ba(4) and 7Ba(24), as well as VBNA forms of 7Ba(24) were used for this study.

**Figure 7:**
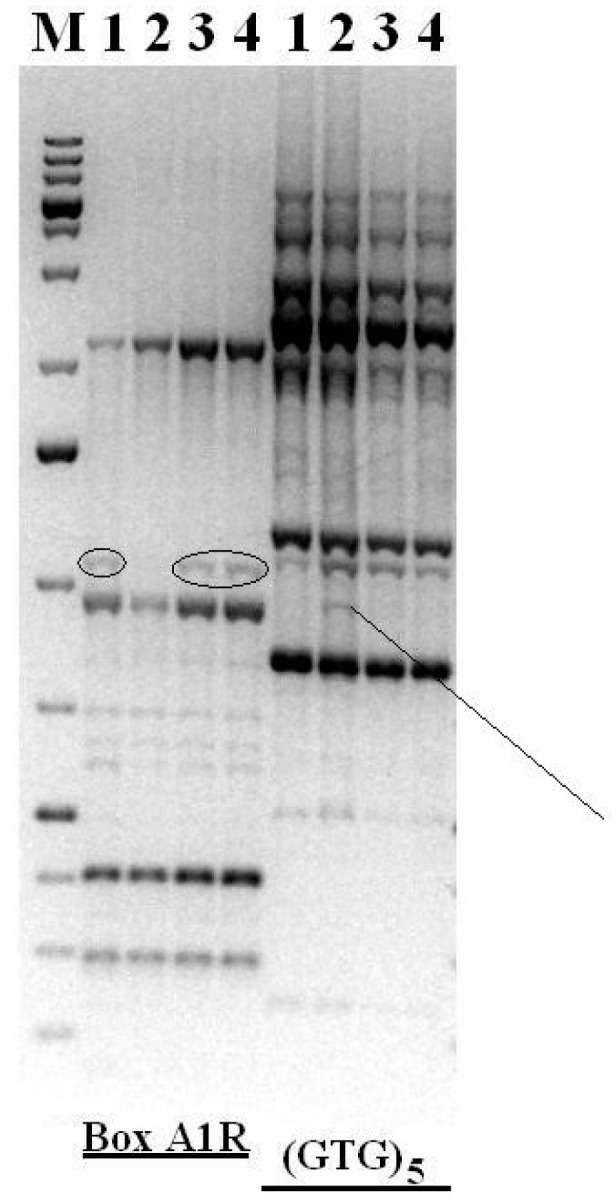
Genomic fingerprint of strain *Rhodococcus* sp. 7Ba with primers BoxA1R and (GTG)_5_. The figure demonstrates changes in the genome by the presence of Box A1R (circles) and (GTG)_5_ (index line) sequence amplification products for different samples. M—GeneRuler 1 kb Plus DNA Ladder, 1—vegetative cells of the 7Ba(4) (vegetative cells of strain 7Ba after dormancy at 4 °C), 2—VBNC cells of the 7Ba(24), 3—vegetative cells of the 7Ba(24) (vegetative cells of strain 7Ba after dormancy at 24 °C), 4—vegetative cells of the 7Ba routinely reseed into laboratory culture.

REP-PCR and subsequent electrophoresis demonstrated that the profiles of genomic fingerprints of all vegetative cell samples of strain 7Ba are similar to each other and differ from VBNC 7Ba(24), although insignificantly (Figure 7). The data obtained allow us to conclude that temporary rearrangements occur in the genome of cells of *Rhodococcus* sp. 7Ba during long-term storage, which are associated with the transition to the VBNC state and are temporary in nature.

## 4. Discussion

We have described forms of long-term (up to 3.5 years) storage of 7Ba cells, which have distinctive morphological properties from vegetative cells, including a prevalence of short cell forms and change (thickening) in the outer cell coatings in the VBNC state, as well as membrane permeabilization. Cells in the VBNC state usually show significant morphological changes, including size reduction and shape changes [21,23,65]. Change in the form of cell wall thickening or thickening of resting cells is a frequent phenomenon. Various resting forms such as spores or cysts have a thick shell that allows them to survive unfavorable conditions. Non-spore-forming bacteria are characterized by the transition of cells into the VBNC state. Ivanushkina et al. report that in contrast to vegetative cells of *Rhodococcus rhodococcus*, VBNC forms are characterized by the shortest coccoid form, reduced cell volume, and absence of division. Significant changes are observed in the cell wall: a decrease in the fluidity of the cytoplasmic membrane, and an increase in the hydrophobicity and adhesiveness of cells [66]. So, changes in cell morphology, thickness of cell covers, and properties of cytoplasmic membrane are adaptation properties characteristic of *Rhodococcus* species. These features ensure cell viability under stress.

Rhodococcus bacteria effectively survive various types of stress. Strain 7Ba shows tolerance to high concentrations of phenol (in our experiments, up to 2 g/L phenol); the cells retained their viability when cultured in such conditions, and the observation was carried out for 14 days. The cell reactivation process is not fully understood; at the moment, it is associated with the involvement of Rpf protein, siderophores, and Autoinducer (AI) [67].

Whole genome analysis showed the presence of genes for phenolic compound degradation, both through the catechol ortho- and meta-pathways of benzene ring cleavage, as well as protocatechate dioxygenase, indicating a pathway of phenolic compound degradation also through protocatecholic acid. Genes for the degradation of various polycyclic and linear compounds such as benzoate, toluene, phenylacetate, xylene, caprolactam, and alkanes were also found. Thus, the analysis of the genome of strain 7Ba indicates a high degradative potential of this strain with respect to a wide range of pollutants.

Analysis of enzyme activity in the cell-free extract indicates that despite the presence of genes for the three phenol degradation pathways, strain 7Ba realizes the catechol ortho-pathway and protocatechuate branch of the β-ketoadipate pathway.

Bacteria in a VBNC state can recover and become suitable for culturing or reactivate when environmental conditions become more favorable, a process known as resuscitation [16].

The ability to grow on 1 g/L phenol of strain 7Ba after the dormancy period is gradually lost, and after 3–4 reseedings, the culture is again able to utilize only 0.5 g/L. At the same time, the newly obtained ability to grow on chlorinated phenol derivatives is retained. Most likely, the genome rearrangements that occur at rest gradually disappear, and the cell passes into a normal vegetative state. Bacteria often possess relatively flexible genome structures and adaptive genetic variants that allow survival under unfavorable growth conditions. Such flexibility plays an important role in ensuring that the most suitable ’genetic’ variants are present in the niche, providing bacteria with an important means of survival in undesirable environments [68,69]. Besides rearranging the genome, the transition to the VBNC state is related to the development of stress and realization of response mechanisms to it; one example is oxidative stress. Oxidative stress is accompanied by the formation of such reactive oxygen species (ROS), so the cells begin to activate the synthesis of antioxidant defense enzymes, including catalase, peroxidase, and superoxide dismutase [70]. Stress also causes activation of chaperone proteins (Hsp33, RidA, and CnoX) and the DNA repair system [71]. Reaction to stress, including reaction leading the cell to the VBNC state, is also associated with changes in the expression of genes responsible for ABC transporters, metabolic enzymes [72], and DNA repair (Nizer 2020). The authors plan further work using transcriptomic and proteomic analysis to clarify these assumptions for strain 7Ba.

Cells after resuscitation from VBNC retained degradation efficiency towards all tested substrates. In natural conditions, particularly soil, cells are often under stress caused by changes in temperature, humidity, pH, oligotrophic nutritional conditions, and so on [67]. Therefore, the VBNC state is probably a universal state for many non-spore-forming bacteria, and the ability of cells to escape from the VBNC state without losing their metabolic potential is an effective way to survive unfavorable conditions.

To date, quite a few studies using Rpf to resuscitate VBNC bacteria are known. Recently, it has been shown to isolate bacteria previously in the VBNC state with the potential for nitrogen removal, salt-resistant phenol degradation, cellulose degradation, and dye degradation by the addition of Rpf [34,37,40,73,74,75]. Studies by Su et al. showed that the addition of Rpf increases the efficiency of phenol biodegradation in effluent under high salinity conditions [75]. Furthermore, the addition of Rpf has been shown to enhance the removal efficiency of phenol, ${\mathrm{PO}}_{4}^{3-}$–P, and total nitrogen in bioreactors [76]. Ye’s group showed that polychlorinated biphenyl (PCB) degradation in the environment can be enhanced by resuscitating PCB-degrading bacteria in the VBNC state [39]. The introduction of Rpf protein not only promotes the bacteria from the dormant state (VBNC), but also enhances their biodegradative potential. In our case, the cells are able to synthesize Rpf themselves, which starts working after the cells are exposed to favorable conditions. 7Ba cells rapidly recover their metabolism and in doing so their metabolic potential against pollutants is enhanced. This is most likely of evolutionary significance—the most adapted and ready to grow rapidly survive.

Phenol and its derivatives are able to be utilized by *Rhodococcus pyridinivorans*, *Rhodococcus opacus*, *Rhodococcus rube*, and *Rhodococcus qingshengii* cells at concentrations ranging from 0.2 to 1.5–2 g/L for free-living cells and up to 3 g/L for those immobilized on various media [77,78,79]. The ability of rhodococci to form VBNC forms is known and successful escape from this state has been studied by various research groups. The addition of Rpf4 protein has been shown to increase proliferation of *R. erythropolis* KB1 cells and improve the recovery of *R. erythropolis* cells from the VBNC state [34]. Addition of exogenous Rpf protein activated cells of the *Rhodococcus biphenylivorans* strain TG9T in the VBNC state, stimulating endogenous expression of Rpf gene orthologs in the cell. The TG9T strain retained its degradative capacity after 90 days of storage under starvation conditions [39]. But there is no study of survival ability under prolonged storage of more than 2 years. Strain 7Ba has the ability to degrade phenol up to 1 g/L as well as chlorophenols up to 0.1 g/L. At the same time, it is able to withstand prolonged storage and does not lose its degradative capacity.

Currently, most researchers agree that the importance of *Rhodococcus* genus representatives as successful biodegraders, platforms for biocatalysis and biosynthesis, is opposed by the fact that these bacteria are not convenient for genetic engineering. This is primarily due to the lack of data reporting on the genetic elements required for such work [80]. However, recently, there has been a growing number of papers on the successful development and application of genetic tools for actinomycetes, particularly *Rhodococcus* [81,82,83]. Thus, there are currently tools available that will allow genetic modification of strain 7Ba. Strain 7Ba, due to its ability to maintain viability for a long time, can be used to enhance and extend its degradative capacity as well as to give it new properties, such as the biosynthesis or bioconversion of compounds required for industry.

## 5. Conclusions

The bacterium *Rhodococcus erythropolis* (*qingshengii*) 7Ba was isolated from contaminated soil. The strain was able to degrade phenol (up to 1 g/L) and chlorophenols (up to 0.1 g/L). Genome analysis showed the presence of genes for the β-ketoadipate pathway of aromatic ring cleavage, and genes for the degradation of alkanes, biphenyls, and others. The cells of strain 7Ba are capable of long-term storage at different temperatures (4 °C and 24 °C), with the cells transitioning to the VBNC state with subsequent recovery of their activity and retention of the ability to degrade aromatic compounds. Analysis of the ultrathin organization of cells of strain 7Ba in different states revealed unique ultrastructural changes characteristic of cells in the VBNC state during storage for up to 3.5 years. The ability of the phenol-degrading strain 7Ba to synthesize Rpf, a factor that allows resuscitation of cells from the VBNC state, provides the possibility of long-term storage of cells without loss of viability, and with retention and even enhancement of biodegradation capacity.

## Figures and Tables

**Table 1:** Sequencing data filtering results.

Parameter	Value
Input Read Pairs	1,349,672
Both Surviving	1,173,100 (86.92%)
Forward Only Surviving	163,148 (12.09%)
Reverse Only Surviving	6314 (0.47%)
Dropped	7110 (0.53%)

**Table 2:** Degradation ability of strain 7Ba depending on storage conditions.

Substrate	7Ba ^1^	7Ba(24) ^2^	7Ba(4) ^3^
Benzoate (0.5 g/L)	+	+	+
Phenol (0.2 g/L)	+	+	+
Phenol (0.5 g/L)	+	+	+
Phenol (1 g/L)	−	+	−
4-chlorophenol (0.1 g/L)	−	+	−
2,4-dichlorophenol (0.1 g/L)	−	+	−
2,4,6-trichlorophenol (0.1 g/L)	−	+	−
Hexadecane (0.1 g/L)	+	+	+

^1^ 7Ba cells routinely reseeded in the laboratory, ^2^ vegetative 7Ba cells after long-term storage at 24 °C, ^3^ vegetative 7Ba cells after long-term storage at 4 °C.

**Table 3:** Assembly metrics of strain 7Ba.

Parameter	Value
Number of contigs	102
Total length of assembly, bp	6,767,064
Longest contig, bp	947,547
Shortest contig, bp	501
N50, bp	310,791
N75, bp	226,191
N90, bp	87,567

## Data Availability

Dataset available on request from the authors.
